# The Genomic Characterization of Equid Alphaherpesviruses: Structure, Function, and Genetic Similarity

**DOI:** 10.3390/vetsci12030228

**Published:** 2025-03-03

**Authors:** Diqiu Liu, Xiaoyang Zhao, Xiaojun Wang

**Affiliations:** State Key Laboratory for Animal Disease Control and Prevention, Harbin Veterinary Research Institute, Chinese Academy of Agricultural Sciences, Harbin 150069, China; zhaoxiaoyang2412@163.com

**Keywords:** equid alphaherpesvirus, genomic structure, function, comparative analysis, future research

## Abstract

Equine herpesvirus 1 (EHV-1), EHV-4, EHV-8, and EHV-9, are classified within the subfamily *Alphaherpesvirinae* and are recognized as causative agents of respiratory, urogenital, and neurological disorders in horses. These viruses, collectively referred to as αEHVs, exhibits both unique and shared characteristics in terms of host interaction, pathogenesis, epidemiology, and immune evasion, which arise from both the identities and discrepancies among respective genomic homologs. The genomic architecture of αEHVs is similar to other members of the same subfamily, such as well-known HSV-1, VZV, and PRV. However, research on the molecular mechanisms underlying αEHV infection and immune response remains significantly less advanced compared to studies on human, porcine, and bovine herpesviruses. This paper systematically describes the genomic structure, function, and genetic similarities of αEHVs and conducts a comparative analysis of selected αEHVs through pairwise sequence alignments of nucleotides and amino acids. This review offers an extensive synthesis of the current understanding related to the study of αEHVs, highlighting the challenges and potential solutions for future research endeavors.

## Simple Summary

Genomic structure, genomic function, and genetic similarity are fundamental components of the field of genomics. Equid herpesviruses in subfamily alphaherpesvirinae include equine herpesvirus 1 (EHV-1), EHV-3, EHV-4, EHV-6, EHV-8, and EHV-9, which are recognized as etiological agents responsible for respiratory, urogenital, and neurological disorders in equine species, exhibiting unique and similar characteristics in infection that are influenced by both the identities and differences among their respective genomic homologs. This review systematically examined and synthesized the existing genomic knowledge on αEHVs, focusing on genomic structure, function, and genetic similarity, and conducted pairwise alignments between each homolog. Furthermore, this study identifies essential challenges encountered during the research process and proposes potential solutions. In the current context, future research should prioritize the exploration of unknown genomic functions and novel transcripts within the αEHV genome.

## 1. Introduction

The order *Herpesvirales* consists of families *Orthoherpesviridae*, *Alloherpesviridae*, and *Malacoherpesviridae*, each comprising viruses associated with distinct hosts. Members of *Herpesviridae* infect mammals, birds, or reptiles, while those of *Alloherpesviridae* infect fish or frogs. *Malacoherpesviridae* viruses infect mollusks [1]. *Orthoherpesviridae* has three subfamilies: *Alphaherpesvirinae*, *Betaherpesvirinae*, and *Gammaherpesvirinae* [2]. The five genera of *Alphaherpesvirinae*, including *Iltovirus*, *Mardivirus*, *Scutavirus*, *Simplex virus*, and *Varicellovirus*, comprise all known equid alphaherpesviruses (αEHVs): EHV-1, EHV-3, EHV-4, EHV-6, EHV-8, and EHV-9. EHV-1 shares a close evolutionary relationship and genomic organization with EHV-3 [3]. EHV-3 is the etiological agent responsible for equine coital exanthema, a typically mild genital infection in horses that is transmitted venereally through semen; however, it has been nearly eradicated [4]. EHV-6 is closely related to EHV-3 from the aspect of equine coital exanthema [5]. EHV-6 and EHV-9 are primarily associated with infections in asses and zebras [6]. This review concentrates on the most prevalent and significant αEHVs (EHV-1, EHV-4, and EHV-8) affecting domestic horses and donkeys [6], which are linked to abortion, respiratory diseases, and neurological disorders.

EHV-1 infection can cause upper respiratory disease in horses, presenting symptoms akin to those caused by equine influenza virus [7,8]. In addition to respiratory symptoms, EHV-1 frequently induces neurological syndromes, including ataxia, muscle paresis, paralysis, bladder atony, recumbency, or death. Additionally, EHV-1 has been associated with uterine or placental damage during early gestation and abortion in the late gestation of mares. Horses across all ages occasionally display the classical sings of myeloencephalitis [9]. Similarly, EHV-4 can cause upper respiratory infections in young horses and can also lead to encephalomyelitis with occasional cases of abortion reported [10,11]. Although these two viruses are closely related, they are distinct entities. They were reclassified by the International Committee on Taxonomy of Viruses [1,12]. Infection by EHV-4 typically stays confined to the upper respiratory system, while EHV-1 infection may become systemic following viremia associated with leukocytes [13].

EHV-8, originally designated as asinine herpesvirus 3 (AHV-3) [14], has been implicated in causing febrile rhinitis in donkeys [15,16,17] and abortion in mares [18]. It is noteworthy that the EHV-8 wh strain was isolated from a horse exhibiting neurological symptoms [19]. Phylogenetically, EHV-8 exhibits a closer relationship to EHV-1 and EHV-9 than to EHV-4 [18,19,20,21].

EHV-9, initially termed gazelle herpesvirus-1, represents the most recent addition to the αEHVs group and was first isolated from Thomson’s gazelles that succumbed to fulminant encephalitis [22,23]. It is closely related to EHV-1 and EHV-8 and exhibits a broad host range encompassing species such as hamsters, goats, dogs, cats, polar bears, zebras, giraffes, and gazelles, with the exception of those exhibiting lethal encephalitis [24,25,26,27,28,29]. In equines, however, the clinical presentation is comparatively mild, characterized by moderate encephalitis with reduced neuronal loss, perivascular cuffing, and gliosis [22].

The primary entry point for these viruses is the mucosal surfaces of the respiratory and urogenital tracts, where they initially manifest clinically and often lead to secondary infections. αEHVs are able to establish lifelong latent infections within the lymph nodes, trigeminal ganglia, and leukocytes; however, the exact primary site of latency remains a topic of ongoing debate. Currently, there is no conclusive evidence regarding the incidence and significance of reactivation from latency of EHV-1 and EHV-4. Nonetheless, anecdotal reports suggest that stressful events such as weaning, castration, or transportation may serve as potential causal factors.

This review offers a systematic summary of the current understanding of the genomic structure, function, and genetic similarity of αEHVs and conducts a comparative analysis through pairwise alignment among EHV-1, EHV-4, EHV-8, and EHV-9. These analyses may facilitate future research endeavors aimed at elucidating the molecular mechanisms underlying the distinct and shared characteristics associated with host interaction, pathogenesis, epidemiology, and immune evasion, as influenced by genetic diversity and identity.

## 2. Genomic Structure

The GenBank accession numbers corresponding to the EHV strains analyzed in this study include EHV-1 strain Ab4 (AY665713), EHV-4 strain NS80567 (AF030027), EHV-8 strain wh (JQ343919), and EHV-9 strain P19 (AP010838), with each genome size as shown in Table 1. The determined genome sizes were consistent with the sequences; however, they are unlikely to accurately reflect the actual sizes. This discrepancy arises because each genome sequence contains numerous direct tandem repeats of short sequences, often arranged in complex configurations that include partial or dispersed repeats. In the case of αEHVs, these reiterations (repeats) frequently displayed variability in size, contributing to the heterogeneity observed in genome sizes. Moreover, the tandem repeats located at the genome termini exhibit a high GC content, similar to that observed in telomeric regions. CpG islands are defined by a high concentration of CG dinucleotides, and their relative scarcity is a well-documented evolutionary phenomenon. This scarcity results from the methylation of cytosine residues, which subsequently undergo spontaneous deamination to form thymine, leading to the formation a TG dinucleotide [30]. This mutation can become fixed in the genome through replication processes. Such mechanisms have been observed in herpesviruses during studies investigating the methylation patterns of latent genomes within proliferating cell populations [31]. A class of tandem repeats has been identified within the genomes of Marek’s disease virus and human herpesviruses (HHVs) 6A, 6B, and 7 [31], which may facilitate the integration of the viral genome into the telomeric regions of host eukaryotic chromosomes [32,33]. However, it remains uncertain whether this mechanism is applicable to αEHVs.

Potential protein-coding regions within the EHV-1 genome were initially identified through the search for ATG-initiated open reading frames (ORFs), with a specific focus on evaluating codon usage preferences and the G + C bias at the third codon position. Additionally, general characteristics of the gene organization were taken into account, such as the limited overlap between ORFs and the positioning of potential polyadenylation signals (AATAAA, ATTAAA, and AGTAAA), which are transcribed as part of 3′-coterminal families. This information pertaining to the EHV-1 genome was subsequently utilized to identify EHV-4 genes in an earlier study.

**Figure 1:**
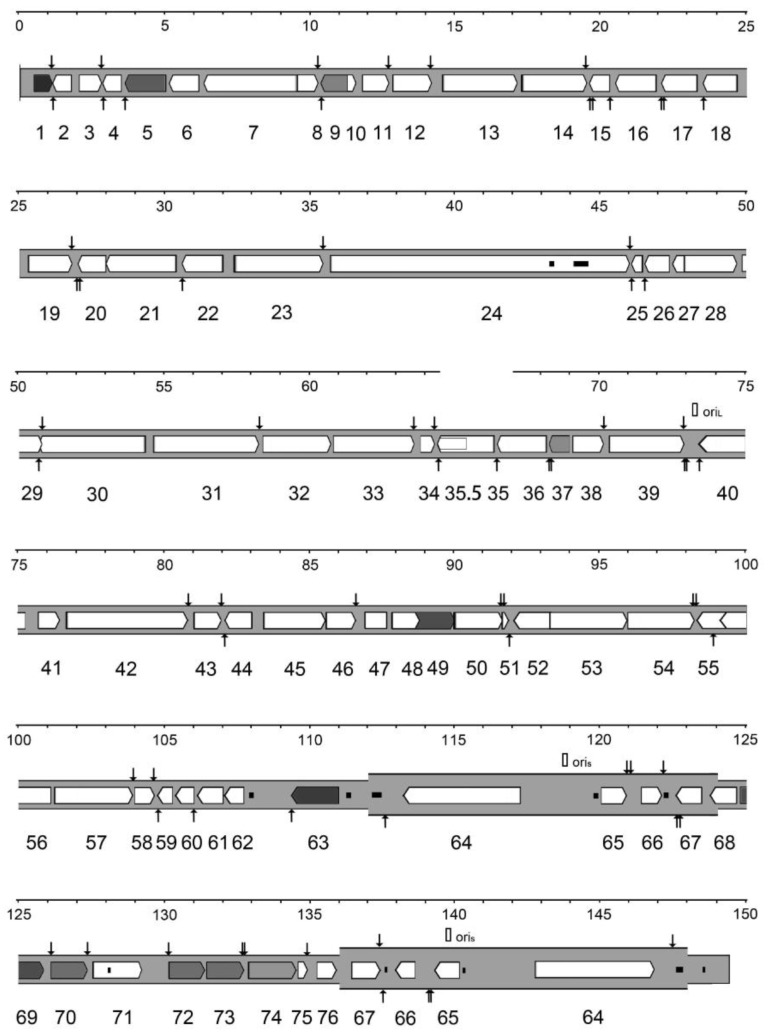
The genomic map of EHV-8. The terminal direct repeat (TR) is shown in a thicker format than the unique region (U). ORFs predicted to encode functional proteins are indicated by arrows (see the key below), with the nomenclature without the ORF prefix given below. The poly(A) sites are indicated by vertical arrows above and below the genome for ORFs oriented toward the right and left, respectively. Reiterated sequences are shown as small filled rectangles, and candidate origins of DNA replication (open squares) are indicated above the genome.

The genome of these viruses comprises unique long (UL), unique short (US), inverted repeat (IR), and terminal inverted repeat (TR) regions. αEHVs are biologically categorized within the *Alphaherpesvirinae* subfamily, which also includes herpes simplex virus type 1 (HSV-1), varicella zoster virus (VZV), and the pseudorabies virus (PRV). Previous studies have detailed the DNA sequences, structural characteristics, and genome mapping of EHV-1 and EHV-4, highlighting their collinear arrangement similarities with HSV-1 and VZV [34,35]. EHV-8 (Figure 1) and EHV-9 (Figure S1) have been identified as possessing a genomic structure analogous to that of EHV-1. In the current study, the four EHV genomes analyzed comprised four distinct components, the dimensions of which are detailed in Table 1. All genomes exhibited highly collinear structures, characterized by a UL region flanked by a short IR linked to a US region, followed by a substantial TR. Notably, with the exception of a single copy of ORF67 in the EHV-4 genome, the other three genomes contained duplicated TRs and IRs for ORF64, ORF65, ORF66, and ORF67. ORF35 was expressed as a full-length mRNA from an upstream promoter, as well as a 3′-coterminal truncated mRNA initiated internally. The protein encoded by the smaller overlapping gene, designated ORF35.5, was identical to the carboxy-terminal portion of the protein encoded by ORF35.

The nomenclature for the ORFs of EHV-8 is derived from that of EHV-1, EHV-4, and EHV-9, with ORFs sequentially numbered from 1 to 76. The ORFs of EHV-8 and EHV-9 are designed in accordance with their homologous counterparts in EHV-1 and EHV-4. In contrast, the nomenclature systems used for other alphaherpesviruses differ from that of the αEHVs, which typically exhibit a dense arrangement of ORFs. Several genomic regions are not anticipated to encode functional proteins; these regions are located at the left and right terminus of αEHVs genome, as well as between ORF62 and ORF63, ORF63 and ORF64, and ORF64 and ORF65. The predicted counts of functional protein-coding ORFs in the four genomes represent the most accurate estimates currently available.

## 3. Gene Function

The potential functions of each protein, as presented and proposed in Table S1, were elucidated through the findings of the current study by integrating known characteristics of the members in *Orthoherpesviridae*, with HSV-1, VZV, and PRV as example.

The current understanding of αEHV genomic function is limited. Existing researches predominantly concentrate on the gene function of EHV-1. EHV-1 utilizes both viral transcription regulatory proteins and host transcription factors to support its infection in vivo and in vitro. The transcriptional regulation of the EHV-1 genome is orchestrated in a temporally controlled cascade, categorized into immediate-early (IE), early (E), and late (L) phases during lytic infection [36]. The sole IE gene, transcribed first, encodes the regulatory protein IEP, which is critical for initiating subsequent viral gene expression. Early genes further prime the cellular environment for viral replication, while late genes, dependent on viral DNA synthesis, encode structural components like glycoproteins (e.g., gB and gC). For example, the EHV-1 IE protein homolog (similar to HSV-1 ICP4) activates E genes, including those involved in DNA replication [37]. The expression of EHV-1 genes over time is intricately regulated by six distinct molecules: the sole IE protein, four early regulatory proteins (EICP0, EICP22, EICP27, and IR2P), and a late ETIF protein [36]. The IE protein enhances the activity of EHV-1 promoters by binding to the consensus sequence 5′-ATCGT-3′, which is found within these promoters. This binding recruits additional TATA-binding proteins (TBP) and transcription factor IIB (TFIIB) to help establish the transcription initiation complex. Conversely, IR2P serves as a negative regulator, diminishing promoter *cis*-activity by depleting the availability of essential cellular transcription factors [38,39], which leads to a significant downregulation in both gene expression and viral titers. The tegument protein ETIF specifically and exclusively activates the IE gene promoter through interactions with transcription factor Oct-1, which targets octamer sequences [40,41]. EICP0 has the capability to independently activate all types of EHV-1 gene promoters by interacting with TBP and TFIIB as well as the simplest promoter only containing TATA site and transcription start site [42,43]. Meanwhile, EICP22 (IR4) [44,45,46] and EICP27 [47,48] each have a minimal trans-activation effect on promoters. However, when they work together, they enhance the activation of the promoter of the IE gene, acting as co-factors that boost IEP activity. EICP22 increases the in vitro host spectrum of EHV-1 and plays an important role in pathogenesis within the murine model. EICP27 synergizes with EICP0 to activate both early and leaky late promoters, and it also directly interacts with TBP. However, EICP22 and EICP0 do not function collaboratively to influence any EHV-1 promoters. Additionally, UL4 interacts with cellular transcription factors, such as the TBP to modulate viral gene expression [49].

The viral DNA polymerase (ORF30) not only facilitates DNA replication but may also engage with cellular RNA polymerase II to enhance transcription processes to *trans*-activate L genes. The polymorphisms in ORF30, being regarded as neuropathogenic markers (e.g., variants harboring A2254/N752 and C2254/H752), have been associated with variations in transcriptional efficiency and clinical outcomes, indicating genotype-specific regulatory differences [50]. The genotype of EHV-1 DNA polymerase (ORF30) [51], specifically variants D752, N752, and H752, correlates with diverse pathogenic profiles [9]; yet, the exact molecular mechanisms remain elusive. Recent genomic studies of EHV-1 outbreaks in Sweden (2012–2021) identified 14 geno-variants, along with new mutations in ORF11 and ORF34 that influence viral fitness [50]. Synonymous mutations in ORF11 and non-synonymous alterations in ORF34 (mutable genomic locus) may modify the secondary structures mRNA or influence protein interactions, impacting transcriptional efficiency [50]. The regulation of EHV-1 transcription is a complex process affected by viral genetic variability, epigenetic alterations, and dynamic protein interactions. Comparative studies with other herpesviruses (e.g., HSV-1) create opportunities to explore unresolved molecular mechanisms. Future inquiries should focus on in vivo models to confirm transcriptional regulators as potential therapeutic targets, especially concerning neuropathogenic strains and approaches to control latency.

Numerous functional proteins require further investigation, identification, and validation. Certain glycoproteins (e.g., gD, gB, gE, and gC) have been shown to facilitate viral entry into host cells, mediate cell-to-cell transportation, elicit protective immune responses, and modulate immune response and are associated with virulence [52,53,54,55,56,57,58,59,60]. The proteins UL43, UL49.5, and UL56 have been shown to downregulate MHC I levels via inhibiting TAP binding [61,62,63,64,65]. EHV-1 UL45 (ORF15) is identified as a virulence factor contributing to neuropathogenesis and influencing viral replication [66,67]. EHV-1 ORF1 and ORF2 exhibit a close relation with virulence during infection in horses [68,69]. US2 (ORF68) modulates virus entry and cell-to-cell spread and relates to virulence [70,71].

EHV-1 expresses two non-coding RNA: latency-associated transcripts (LATs) and IR3. Latency is characterized by the suppression of lytic gene transcription and maintenance of the viral genome via LATs [72,73]. In murine models, EHV-1 reactivation from latency following corticosteroid administration correlated with transient viremia and respiratory shedding, underscoring the reversibility of latency [74]. LATs play a crucial role in establishing latent EHV infections within the host. IR3 suppresses the expression of IE gene, thereby influencing the biological characteristics and virulence of EHV-1. The IR3 transcript of equine herpesvirus-1 (EHV-1) contains 117 nucleotides that are antisense to the IE mRNA, indicating a potential regulatory function [75,76,77].

It is plausible that some of these ORFs do not encode functional proteins, while others may have been overlooked. These ORFs are small, spliced, weakly conserved, or entirely non-conserved and use non-classical initiation codons (i.e., non-ATG), a phenomenon proposed in human herpesvirus (e.g., HSV-2 UL16) [78]. Translational initiation may take place from a codon that is not ATG, which has been demonstrated to serve as an initiation codon in eukaryotic systems [79,80]. Furthermore, although not yet identified, overlapping ORFs may exist within αEHVs genomes. Notably, two ORFs have been identified within the intron located in the 5′ untranslated region (UTR) of the IE gene [81].

## 4. Genetic Similarity

EHV-1 exhibits the largest genome among the four viruses. Furthermore, a homology analysis of the nucleotide and amino acid sequences was conducted for each gene common to the four viruses (Table S2), as any genetic diversity is likely to result in distinct characteristics in the interaction of αEHVs. The pairwise alignment between homologous ORFs and their products (amino acid sequence), annotated in EHV-1, EHV-4, EHV-8, and EHV-9, were performed using the Clustal W algorithm of the MegAlign platform with DNAstar (version 7.1) software.

### 4.1. Conserved ORFs in Orthoherpesviridae

Members of the *Orthoherpesviridae*, within the order *Herpesviriales*, possess 44 shared genes (referred to as core ORFs, Table S1), which are seemingly inherited from a common ancestor [82]. This genetic composition highlights a greater divergence within the *Herpesviridae* family compared to the *Alloherpesviridae* family, which contains only 12 core genes [31]. These genes encompass the majority of functional categories outlined in Table S1, including those associated with nucleotide metabolism, DNA replication, capsid structure, and virion morphogenesis.

The ORF44/47 encoding the DNA packaging terminase subunit 1 exhibits a high degree of conservation across members of the order *Herpesvirales* [83]. Conversely, the ORF encoding the DNA packaging terminase subunit 2 (ORF32) is conserved within the *Orthoherpesviridae* but demonstrates only limited similarity to the core gene (ORF47) in cyprinid herpesvirus, with no analogous sequences identified in other alloherpesviruses [31]. Notably, the conserved region encompasses a cysteine-rich motif, which is ubiquitous among *Orthoherpesviridae* members and may function as a metal ion-binding domain [84,85,86].

### 4.2. Conserved Genes in Subfamily Alphaherpesvirinae

In the *Alphaherpesvirinae* subfamily, a total of 24 conserved genes were identified except for 44 core genes. Of these, 44 core genes were inherited from the common ancestor shared by the α-, β-, and γ-subfamilies of *Orthoherpesviridae*, while these 24 genes were inherited from an ancestor specific to the *Alphaherpesvirinae* subfamily [82], as indicated in Table S1 using orthologs. This phylogenetic classification suggests that specific core or orthologous genes may have been lost in certain lineages. In the case of αEHVs, 11 viral glycoproteins [87], including gB, gC, gD, gE, gG, gH, gI, gK, gL, gM, and gN, are conserved when compared to other alphaherpesviruses. The glycoprotein gp2 (gJ) is encoded by ORF71 exclusively in αEHVs and is located within the US genomic segment [88,89,90]. EHV-1 strain Kentucky A (KyA) harbors a 1242 bp deletion and encodes a truncated gp2 of only 383 amino acids [89,90], in contrast to 797 and 791 amino acids in the pathogenic strains Ab4 and RacL11, respectively. As regulatory proteins, ORF64 (IEP), truncated ORF64 (IR2P), ORF12 (ETIF), and ORF5 (EICP27) are conserved in *Alphaherpesvirinae*. The remaining eight genes—ORF2, ORF3, ORF4, ORF34, ORF63 (EICP0), 65 (EICP22), ORF67 and ORF76—are not conserved in this subfamily. Of the 76 ORFs, there is no knowledge to date about potential functions associated with ORF4, ORF58, ORF60, ORF66, and ORF75.

### 4.3. Orthologs in HSV-1 and VZV

The αEHVs exhibit 71 and 70 orthologs in common with HSV-1 and VZV, respectively (Table S1). The degree of genome collinearity between αEHVs and either HSV-1 or VZV is comparable to that observed among αEHVs themselves, characterized by extensive blocks that are rearranged in terms of order and orientation relative to one another. These findings underscore the close evolutionary relationship among αEHVs. It is important to note that while αEHVs are considered to demonstrate a pattern of recent coevolution with their hosts, this does not necessarily imply cospeciating with their hosts, nor with HSV-1 and VZV [34,35]. Therefore, within the *Alphaherpesvirinae* subfamily, αEHVs, HSV-1, and VZV exhibit a closer phylogenetic relationship to one another than to their respective host species, suggesting that one of these lineages likely evolved through an interspecies transfer event. Future and ongoing studies may be informed and guided by the relevant information associated with orthologs from HSV-1 and VZV.

### 4.4. Fragmented ORF

EHV-1 and EHV-4 have been documented to possess a single fragmented open reading frame (ORF 44/47), while the EHV-8 and EHV-9 equivalents have been annotated in GenBank, albeit containing an intergenic region that is highly conserved among αEHVs, which are homologous to the two exons of HSV-1 UL15, which is expressed as a spliced mRNA.

### 4.5. UL24 Protein Family in Orthoherpesviridae

The UL24 protein family is conserved across all three subfamilies of the *Orthoherpesviridae* [91]. This conservation suggests the existence of the same ancestor gene that dates back at least 180 million years, prior to the emergence of the three subfamilies [92,93]. The UL24 family encodes a variety of potential PD-(D/E)XK endonucleases, characterized by the presence of signature motifs II, III, and IV, which are part of a broad and diverse superfamily of restriction endonucleases and recombinases [94,95]. The UL24 protein can inhibit the innate immune response of the host through its interaction with various immune signaling pathways (e.g., RIG-I, cGAS-STING) involved in a series of cellular factors, including p65, p50, IRF7, ISG20, IL-8, OASL, IL-β, and ZCCHZ3, during the course of a viral infection. This multifaceted role not only allows the virus to evade the initial defenses of the host immune system but also contributes significantly to the ability of herpesvirus to replicate and become pathogenic in the later stages of the infection [91,95]. UL24 is crucial for the herpesvirus lifecycle, enhancing both its proliferation and its capacity to cause disease in the category of *Orthoherpesviridae*.

In HHVs, the UL24 protein induces cell cycle arrest by inactivating the cyclin B/cdc2 complex and may also function as a minor tegument component that is weakly associated with capsids [96]. The pervasive presence of the UL24 the *Orthoherpesviridae* indicates its essential function in the viral life cycle, particularly in processes such as membrane fusion and replication [95]. This role is evidenced in tissue culture by the dispersal and redistribution of the nucleolar protein (NPM1) [96,97,98]. Currently, there are only two documented studies concerning UL24 in EHV-1. One study, utilizing a mouse model, identified UL24 as a determinant of neuropathogenicity, while another study provided sequence data for its homolog from Brazilian isolates of EHV-1 [99,100]. In equid herpesviruses, the biological function of the UL24 protein warrants further investigation, and elucidating the associated molecular mechanisms may be facilitated by leveraging research conducted on other herpesviruses within the same family. EHV-1 UL24/TK (head to head located genome) deletion may be the prospective target to break latent infection and pave the novel way to develop vaccine and antiviral reagents aiming at the infection of αEHVs [101].

## 5. Problems and Possible Solutions

Currently, there are no immortalized cell lines derived from the respiratory and nervous systems of equids, which poses significant challenges to understanding the molecular mechanisms underlying the pathogenesis of αEHVs in vivo. In the context of αEHV research, commonly used cell lines such as RK13, BHK-21, CHO, and MDBK can support the replication of EHV-1, EHV-3, EHV-8, and EHV-9 in vitro; however, these cell lines exhibit considerable genetic diversity when compared to equine cells. Fetal horse kidney cells (FHK) and equid dermis cells (ED) are utilized in certain studies related to EHV-4, albeit within a limited number of passages.

The transformation of FHK cells with primate genes, including large T antigen of SV40 (LTA), results in their immortalization, allowing them to be employed for the isolation and propagation of equine herpesvirus [102,103,104]. The fields of genomics, transcriptomics, proteomics, and metabolomics in equids are currently underdeveloped, resulting in a lack of comprehensive information regarding gene function. This shortcoming poses a significant barrier to advancing our understanding of the molecular interactions between EHVs and horses. Consequently, the equine telomere reverse transcriptase (eTERT) gene may be viewed as a potential catalyst for the development of immortalized equine cell lines and oncogenic genes associated with equine adenovirus, similar to the established immortalized human cell lines. The establishment of such cell lines could greatly enhance research efforts focused not only on EHVs but also on other equine pathogens.

## 6. Conclusions and Prospects

This review provides a systemic summary of the genomic information of αEHVs, focusing on their structural and functional characteristics, highlighting the identity and diversity among homologs through comparative alignment. This review represents the initial investigation of a collection of closely related αEHV genomes and aims to enhance the understanding of αEHVs within the *Orthoherpesviridae* family to match the depth of knowledge available for the far more thoroughly researched Herpesviridae family. Similar to what is becoming evident with human herpesviruses (e.g., HSV-1 and VZV), the genomic characteristics of αEHVs will provide a foundation for forthcoming research on the pathogenesis and immune evasion mechanisms of αEHVs and for efforts focused on vaccine and therapeutics, diagnostics, and epidemiological advancements.

In the context of current biological research, initial investigations might prioritize targeting αEHVs genes labeled as “unknown” and “possibly” in terms of function, as indicated in Table S1. The observed discrepancies between homologs may facilitate the establishment of differential diagnoses. Investigating, detecting, identifying, and confirming novel transcription units within the αEHV genome represent significant research avenues, particularly because certain AATAAA sequences have not been annotated as poly(A) signal sites in GenBank data. These AATAAA motifs likely suggest the presence of previously uncharacterized transcripts. Also, future research should aim to identify and confirm non-essential genes involved in viral replication in vitro. By targeting these regions for genetic editing, the genome of αEHVs could be optimized as a live vector for the delivery of heterologous antigens, thereby enhancing strategies for the prevention and treatment of infectious diseases in equids. In summary, this review aims to advance research on αEHVs within the current context.

## Figures and Tables

**Table 1:** Genomic arrangement and size of αEHVs in subfamily *Alphaherpesvirinae*.

Name	Size (bp)					G + C (%)	Total Numbers of ORFs				
	Genome	U_L_^a^	U_S_^b^	IR ^c^	TR ^d^		Genome	U_L_	U_S_	IR	TR
EHV-1	150,224	112,935	11,861	12,714	12,714	56.67	80	62	10	4	4
EHV-4	145,597	112,452	12,789	10,179	10,179	50.46	79	62	10	4	3
EHV-8	149,332	113,341	89,403	11,969	11,969	54.36	80	62	10	4	4
EHV-9	148,371	112,681	11,998	11,846	11,846	56.10	80	62	10	4	4

^a^, long unique region; ^b^, short unique region; ^c^, inverted repeat; ^d^, terminal inverted repeat.

## Data Availability

The original contributions presented in this study are included in this article and supplementary material. Further inquiries can be directed to the corresponding authors.

## Supplementary Materials

The following supporting information can be downloaded at: https://www.mdpi.com/article/10.3390/vetsci12030228/s1, Figure S1: The genomic map of EHV-9. The terminal direct repeat (TR) is shown in a thicker format than the unique region (U). ORFs predicted to encode functional proteins are indicated by arrows (see the key below), with the nomenclature without the ORF prefix given below. The poly(A) sites are indicated by vertical arrows above and below the genome for ORFs oriented toward the right and left, respectively. Reiterated sequences are shown as small filled rectangles, and candidate origins of DNA replication (open squares) are indicated above the genome; Table S1: Features of functional protein-coding regions of EHVs in *Alphaherpesvirinae*; Table S2: Alignment of protein-coding regions of EHVs in *Alphaherpesvirinae* (%).
