# Supplementary material for: The Genomic Characterization of Equid Alphaherpesviruses: Structure, Function, and Genetic Similarity
Source: Vet Sci. 2025 Mar 3;12(3):228. doi: 10.3390/vetsci12030228 (PMC11945689; doi:10.3390/vetsci12030228)
Supplement: Supplementary file 1 [file vetsci-12-00228-s001.zip › Figure S1-EHV-9 genome-Revised.pdf]

Figure S1

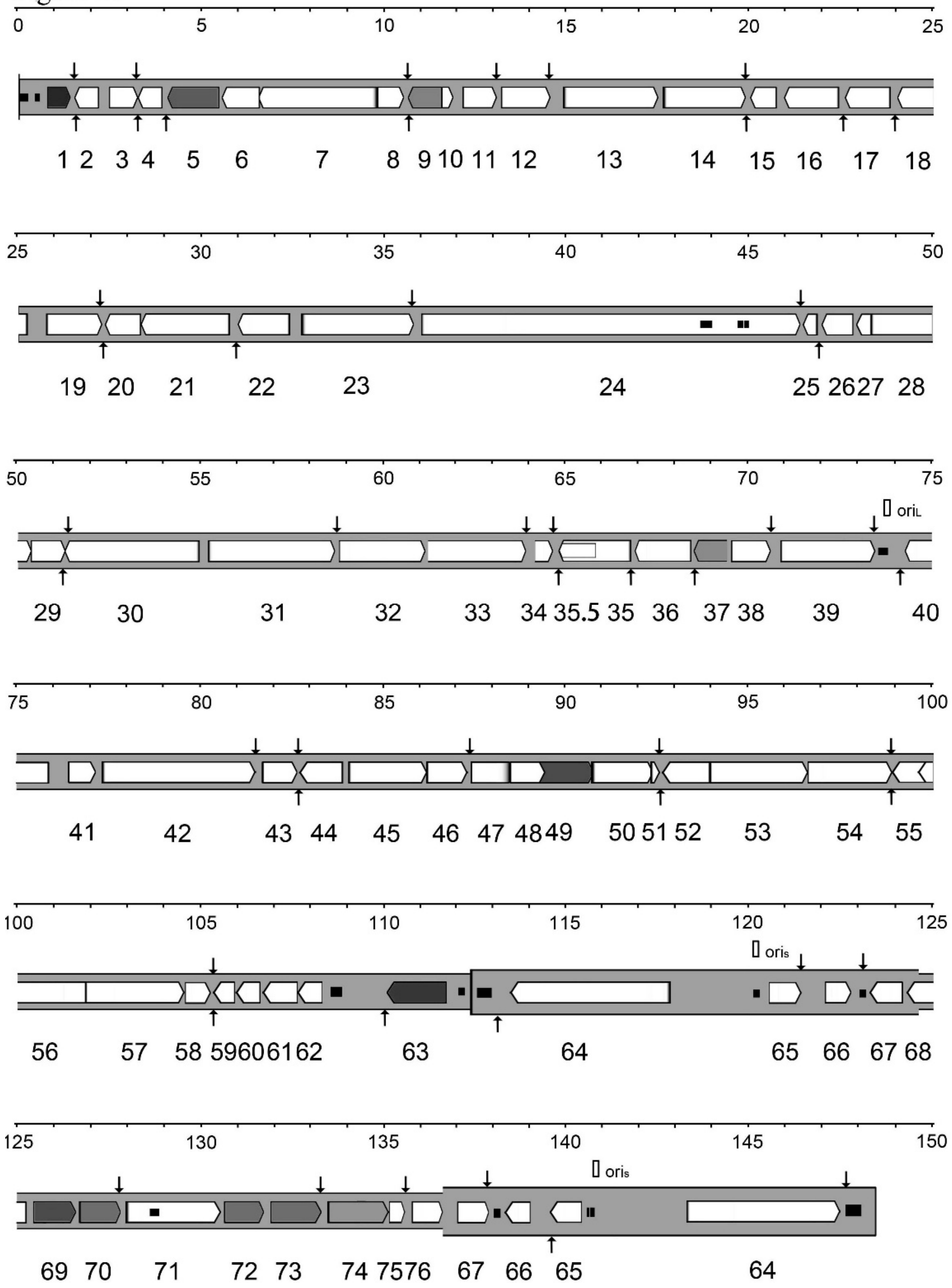

Figure S1.The genomic map of EHV-9. The terminal direct repeat (TR) is shown in a thicker format than the unique region (U). ORFs predicted to encode functional proteins are indicated by arrows (see the key below), with the nomenclature without the ORF prefix given below. The poly(A) sites are indicated by vertical arrows above and below the genome for ORFs oriented toward the right and left, respectively. Reiterated sequences are shown as small filled rectangles and candidate origins of DNA replication (open squares) are indicated above the genome.
