# Supplementary material for: The Genomic Characterization of Equid Alphaherpesviruses: Structure, Function, and Genetic Similarity
Source: Vet Sci. 2025 Mar 3;12(3):228. doi: 10.3390/vetsci12030228 (PMC11945689; doi:10.3390/vetsci12030228)
Supplement: Supplementary file 1 [file vetsci-12-00228-s001.zip › Supplementary Table S1-revised.pdf]

Table S1. Features of functional protein-coding regions of EHVs in *Alphaherpesvirinae*

ORF[a]	Homologue		Product&Characteristics	Function	Gene conservation[c,d]	Orientation[b]
	HSV-1	VZV				
1	UL56	0	Membrane protein UL56; type II membrane protein	**Possibly** responsible to vesicular trafficking	Ortholog	L
2	-	1	Membrane protein V1; type II membrane protein; homodimer; enclosed into virion.	**Possibly** changing the cell adhesion molecules in infected cells		R
3	-	2	tegument protein being myristylated	**Possibly** envelope-associated		L
4	UL55	3	Nucleus protein	**Unknown**		R
5	UL54	4	EICP27; RNA-binding protein; multifunctional expression regulator	Gene transcription regulation; RNA metabolism & shuttles between nucleus and cytoplasm; impeding pre-mRNA splicing; transporting mRNA from nucleus exerts most effects post-transcriptionally;	core	R
6	UL53	5	gK; Type III membrane protein; IV transmembrane domains	Virion morphogenesis; membrane fusion	Ortholog	R
7	UL52	6	Subunit of helicase-primase complex	Genomic DNA replication;	core	R
8	UL51	7	Tegument protein UL51	Virion morphogenesis	core	L
9	UL50	8	Deoxyuridine triphosphatase	Nucleotide metabolism	core	R
10	UL49A	9A	gN; type I membrane protein; contains a signal peptide; complexed with envelope glycoprotein M	Virion morphogenesis; membrane fusion	core	L
11	UL49	9	Tegument protein VP22	Virion morphogenesis; **possibly** RNA transport to uninfected cells	Ortholog	L
12	UL48	10	ETIF; trans-activating tegument protein VP16	Gene transcription regulation; virion morphogenesis; transactivates immediate early gene	Ortholog	L
13	UL47	11	Tegument protein VP13/14	**Possibly** gene transcription regulation; modulates and cooperate tegument protein VP16; RNA-binding protein	Ortholog	L
14	UL46	12	Tegument protein VP11/12	**Possibly** gene regulation; modulates transactivating tegument protein VP16	Ortholog	L
15	UL45	-	Membrane protein UL45	**Possibly** membrane fusion; type II membrane protein; tegument-associated	Ortholog	R

Table S1. Features of functional protein-coding regions of EHVs in *Alphaherpesvirinae*

ORF[a]	Family	Homologue		Product& Characteristics	Function	Gene conservation[c,d]	Orientation[b]
		HSV-1	VZV				
16		UL44	14	gC; type I membrane protein; contains a signal peptide; binds cell surface heparan sulphate; binds complement C3b to block neutralization	Cell attachment	Ortholog	R
17		UL43	15	Envelope protein UL43; type III membrane protein; 11 transmembrane domains	**Possibly** membrane fusion	core	R
18		UL42	16	DNA polymerase processivity subunit; dsDNA-binding protein;	DNA replication	core	R
19		UL41	17	Tegument host shutoff protein; mRNA-specific RNase	Cellular mRNA degradation	Ortholog	L
20		UL40	18	Ribonucleotide reductase subunit 2	Nucleotide metabolism	core	R
21		UL39	19	Ribonucleotide reductase subunit 1	Nucleotide metabolism	core	R
22		UL38	20	Capsid triplex subunit 1; complexed 1:2 with capsid triplex subunit 2 to connect capsid hexons and pentons; VP19C	Capsid morphogenesis	core	R
23		UL37	21	Tegument protein UL37; complexed with large tegument protein	Virion morphogenesis	core	L
24		UL36	22	Large tegument protein; complexed with tegument protein UL37; ubiquitin-specific protease N-terminal region; VP1/2	Capsid transport; capsid morphogenesis	core	L
25		UL35	23	Small capsid protein; located externally on capsid hexons	**Possibly** capsid transport; capsid morphogenesis	core	R
26		UL34	24	Nuclear egress membrane protein; type II membrane protein; interacts with nuclear egress lamina protein	Nuclear egress	core	R
27		UL33	25	DNA packaging protein; interacts with DNA packaging terminase subunit 2	DNA enclosing	core	R
28		UL32	26	DNA packaging protein	DNA enclosing; **possibly** capsid transport	core	L
29		UL31	27	Nuclear egress lamina protein; interacts with nuclear egress membrane protein	Nuclear egress	core	L
30		UL30	28	DNA polymerase catalytic subunit	DNA replication	core	R
31		UL29	29	Single-stranded DNA-binding protein; contains a zinc-finger	DNA replication; **possibly** gene regulation	core	L
32		UL28	30	DNA packaging terminase subunit 2	DNA encapsidation	core	L

(Continued on following page)

Table S1. Features of functional protein-coding regions of EHVs in *Alphaherpesvirinae*

ORF[a]	Homologue		Protein& Characteristics	Function	Gene conservation[c,d]	Orientation[b]
	HSV-1	VZV				
33	UL27	31	gB; type I membrane protein; contains a signal peptide; possible membrane fusogen; binds cell surface heparin sulphate	Cell-to-cell spread; cell entry	core	L
34	-	32	Protein V32	**Possibly** playing a role in replication to high titers		L
35	UL26	33	Capsid maturation protease; serine protease (N-terminal region); minor scaffold protein (remainder of protein, clipped near C terminus)	Capsid morphogenesis	core	R
35.5	UL26.5	33.5	Capsid scaffold protein; clipped near C terminus	Capsid morphogenesis	core	R
36	UL25	34	DNA packaging tegument protein; located on capsid near vertices; **possibly** stabilizes the capsid and retains the genome	DNA encapsidation	core	R
37	UL24	35	Nuclear protein	Gene expression regulation	core	R
38	UL23	36	Thymidine kinase (TK)	Nucleotide metabolism	core	L
39	UL22	37	gH; type I membrane protein; contains a signal peptide; possible membrane fusogen; complexed with envelope glycoprotein L	Cell entry; cell-to-cell spread	core	L
40	UL21	38	Tegument protein UL21; interacts with microtubules	Virion morphogenesis	Ortholog	R
41	UL20	39	Envelope protein UL20; type III membrane protein; 4 transmembrane domains	Virion morphogenesis; membrane fusion	Ortholog	L
42	UL19	40	Major capsid protein; 6 copies form hexons, 5 copies form pentons	Capsid morphogenesis	core	L
43	UL18	41	Capsid triplex subunit 2; complexed 2:1 with capsid triplex subunit 1 to connect capsid hexons and pentons	Capsid morphogenesis	core	L
47/44	UL15	45/42	DNA packaging terminase subunit 1 contains an ATPase domain	DNA enclosing	core	R
45	UL17	43	DNA packaging tegument protein; capsid-associated	DNA enclosing; capsid transport	core	L
46	UL16	44	Tegument protein	**Possibly** virion morphogenesis	core	L
48	UL14	46	Tegument protein	Virion morphogenesis	core	L
49	UL13	47	Tegument serine/threonine protein kinase	Protein phosphorylation	core	L

(Continued on following page)

Table S1. Features of functional protein-coding regions of EHVs in *Alphaherpesvirinae*

ORF[a]	Homologue		Protein& Characteristics	Function	Gene conservation[c,d]	Orientation[b]
	HSV-1	VZV				
50	UL12	48	Deoxyribonuclease	DNA processing	core	L
51	UL11	49	Myristylated tegument protein; envelope-associated	Virion morphogenesis	core	L
52	UL10	50	gM; type III membrane protein; 8 transmembrane domains; complexed with envelope glycoprotein N	Virion morphogenesis; membrane fusion	core	R
53	UL9	51	DNA replication origin-binding helicase	DNA replication	core	L
54	UL8	52	Helicase-primase subunit	DNA replication	core	L
55	UL7	53	Tegument protein	Virion morphogenesis	core	R
56	UL6	54	Capsid portal protein; dodecamer located at one capsid vertex in place of a penton	DNA encapsidation	core	R
57	UL5	55	Helicase-primase helicase subunit	DNA replication	core	L
58	UL4	56	Nuclear protein UL4; colocalizes with regulatory protein ICP22 and nuclear protein UL3 in small, dense nuclear bodies	**Unknown**	Ortholog	L
59	-	57	Protein V57	**Possibly** virion morphogenesis	Ortholog	R
60	UL3	58	Nuclear protein UL3; colocalizes with regulatory protein ICP22 and nuclear protein UL4 in small, dense nuclear bodies	**Unknown**	Ortholog	R
61	UL2	59	uracil-DNA glycosylase	DNA repair	core	R
62	UL1	60	gL; contains a signal peptide; complexed with envelope glycoprotein H	Cell entry; cell-to-cell spread	core	R
63	IE110	61	EICP0; contains a RING finger; disrupts ND10; proteasome-dependent degradation of several cellular proteins	Gene regulation; cellular protein degradation; Neuropathogenicity determinant		R
64	IE175	62	IE; transcriptional regulator ICP4	Gene regulation	Ortholog	R
65	US1	63	EICP22; required for expression of a subset of late genes	Gene regulation; cell cycle regulation		L

(Continued on following page)

Table S1. Features of functional protein-coding regions of EHVs in *Alphaherpesvirinae*

ORF[a]	Homologue		Protein& Characteristics	Function	Gene conservation[c,d]	Orientation[b]
	HSV-1	VZV				
66	US10	64	Virion protein US10	**Unknown**	Ortholog	L
67	-	-	Virion protein V67; colocalizes with nuclear lamins	Influencing location of PK and virus growth at elevated temperature; a major determinant of virulence		R
68	US2	-	Virion protein US2; **possibly** envelope- associated; interacts with cytokeratin 18	**Unknown**	Ortholog	R
69	US3	66	Serine/threonine protein kinase; tegument protein; phosphorylates nuclear egress lamina protein; mediates phosphorylation of HDAC1and HDAC2 and other cellular and viral proteins;	Protein phosphorylation; apoptosis; nuclear egress	Ortholog	L
70	US4	-	gG; type I membrane protein; contains a signal peptide;	Cell-to-cell spread	Ortholog	L
71	US5	-	gJ; type I membrane protein; contains a signal peptide	Possible determinant of respiratory virulence	Ortholog	L
72	US6	-	gD; type I membrane protein; contains a signal peptide; binds cell surface receptors	Cell attachment	Ortholog	L
73	US7	67	gI; type I membrane protein; contains a signal peptide; complexed with envelope glycoprotein E to form an Fc-receptor	Cell-to-cell spread	Ortholog	L
74	US8	68	gE; type I membrane protein; contains a signal peptide; complexed with envelope glycoprotein I to form an Fc-receptor	Cell-to-cell spread	Ortholog	L
75	US8A	-	Membrane protein US8A; type I membrane protein	**Unknown**	Ortholog	L
76	US9	65	Membrane protein US9; type I membrane protein; tegument-associated; localizes envelope proteins	Axonal transport		L

[a]ORFs are listed in a default order, basically in relation to their arrangement in EHV-1, EHV-4, EHV-8, and EHV- 9.

[b]ORFs are oriented rightward (R) and leftward (L) in the four herpesviruses.

[c]Orthologs, orthologous genes in alphaherpesviruses.

[d]Core genes, inherited from an ancestor of alpha-, beta- and gammaherpesviruses
