# Supplementary material for: The Genomic Characterization of Equid Alphaherpesviruses: Structure, Function, and Genetic Similarity
Source: Vet Sci. 2025 Mar 3;12(3):228. doi: 10.3390/vetsci12030228 (PMC11945689; doi:10.3390/vetsci12030228)
Supplement: Supplementary file 1 [file vetsci-12-00228-s001.zip › Supplementary Table S2-revised.pdf]

Table S2. Alignment of protein-coding regions of EHVs in *Alphaherpesvirinae* (%)

ORF	Protein						DNA					
	1/4	1/8	1/9	4/8	4/9	8/9	1/4	1/8	1/9	4/8	4/9	8/9
1	67.3	91.1	95.5	63.8	67.8	91.6	69.1	87.7	94.7	69.1	70.3	89.2
2	71.7	86.3	93.1	69.1	72.5	89.2	72.9	88.3	93.8	72.6	73.6	90.7
3	74.6	90.7	92.6	74.6	76.6	94.2	73.0	90.4	91.2	74.4	74.8	93.0
4	87.0	92.5	93.0	87.0	88.5	95.0	81.1	92.2	92.2	81.1	81.6	94.0
5	77.1	93.4	94.7	76.4	77.3	95.5	75.9	91.2	92.7	76.3	76.2	92.4
6	89.5	98.0	98.5	88.9	89.5	98.0	82.4	93.8	95.3	82.1	82.7	95.0
7	80.2	93.1	95.2	79.4	80.7	95.5	79.1	92.0	93.8	78.3	79.2	94.7
8	83.5	96.3	96.3	83.5	84.0	97.6	81.1	92.1	93.8	81.4	81.8	95.8
9	77.6	92.3	93.9	77.3	78.2	96.3	79.7	92.3	93.8	79.0	79.3	95.7
10	88.0	95.0	95.0	89.0	90.0	98.0	87.1	93.7	96.4	88.1	88.8	96.7
11	78.0	92.1	93.4	77.6	80.6	95.4	78.3	91.9	92.9	77.7	78.6	94.4
12[b]	86.6	92.4	97.3	86.6	87.5	93.3	80.6	89.0	94.9	83.3	81.2	89.5
13	84.8	84.8	93.8	87.0	85.6	85.9	80.7	82.3	92.2	81.9	80.0	83.1
14	79.0	92.7	94.5	80.0	79.6	95.4	75.7	91.0	92.5	76.2	76.3	93.6
15	68.8	85.5	86.2	71.2	72.1	92.1	72.6	88.6	89.0	73.9	74.9	93.7
16	81.4	94.9	96.4	81.2	80.6	95.3	77.6	93.7	94.8	77.8	77.5	95.3
17	84.3	96.3	96.0	83.5	84.3	97.8	79.5	93.2	93.9	79.0	79.8	94.3
18	83.5	97.0	96.5	83.5	83.7	98.0	78.6	93.7	95.1	77.4	78.5	94.9
19	84.5	94.4	96.0	83.6	85.1	97.6	79.7	92.0	93.1	80.0	80.1	96.0
20	89.7	96.3	98.1	88.4	89.4	96.9	81.8	93.2	94.7	81.1	81.5	95.4
21	88.3	96.6	96.2	89.4	88.8	97.5	81.6	94.0	95.5	81.9	82.3	94.8
22	91.8	95.3	96.6	91.6	92.0	98.3	82.7	92.2	94.3	82.9	82.9	95.5
23	89.1	93.6	98.1	88.7	89.2	94.3	80.7	86.1	94.2	80.9	80.4	87.4
24	76.5	91.6	96.6	75.9	77.0	93.0	75.2	90.0	94.3	75.6	75.7	91.7
25	88.2	99.2	96.6	89.1	89.1	97.5	82.2	95.6	98.3	84.4	83.3	95.3
26	90.9	94.9	94.5	90.9	91.6	98.2	82.1	92.6	93.5	81.6	81.8	97.1

(Continued on following page)

Table S2. Alignment of protein-coding regions of EHVs in *Alphaherpesvirinae* (%)

ORF	Protein						DNA					
	1/4	1/8	1/9	4/8	4/9	8/9	1/4	1/8	1/9	4/8	4/9	8/9
27	79.1	90.0	94.2	76.9	79.1	94.6	79.3	91.1	93.1	78.9	79.0	95.4
28	80.7	90.0.	92.7	79.5	80.3	95.0	78.3	90.9	92.6	78.2	79.0	95.4
29	91.7	98.2	98.5	91.1	92.0	98.5	82.8	95.6	95.5	82.3	83.4	96.2
30	88.3	96.1	96.7	87.6	88.3	97.2	81.7	92.9	94.8	81.4	82.0	95.1
31	90.6	97.6	98.3	90.2	90.7	97.9	81.4	94.0	94.5	81.3	81.6	95.6
32	88.0	96.6	97.8	87.5	88.1	96.8	81.9	94.5	95.8	81.1	81.8	96.1
33	89.6	97.6	98.2	93.9	92.8	97.5	83.2	94.9	96.3	84.9	84.4	96.0
34	73.0	89.3	93.7	73.6	73.4	92.4	74.8	91.2	92.9	76.5	76.4	94.2
35.5	83.9	96.4	96.4	82.7	82.4	96.4	79.9	93.5	93.9	78.7	78.7	95.4
35	87.8	97.4	96.7	90.3	96.1	97.3	81.5	94.1	94.8	80.8	80.7	96.1
36	90.3	96.1	97.3	90.6	91.0	97.1	80.3	92.1	93.8	80.4	80.4	94.0
37	83.5	93.5	95.9	81.7	82.5	94.7	82.8	93.4	95.4	81.4	82.3	94.6
38	88.9	97.2	98.6	87.8	88.4	98.0	83.2	94.7	95.6	83.2	82.9	96.0
39	86.1	96.0	96.2	86.3	85.8	96.6	80.9	92.5	93.4	81.2	80.8	94.9
40	86.6	97.2	97.4	86.2	86.8	97.7	79.7	93.3	94.0	78.6	79.6	95.5
41	86.5	97.5	97.5	86.1	86.1	97.1	86.7	95.4	96.0	85.7	85.6	95.7
42	96.4	99.0	99.1	95.9	95.8	99.1	84.2	93.9	95.1	83.6	83.6	95.4
43	94.6	98.4	97.8	94.3	93.6	98.7	84.1	93.3	95.2	83.4	83.5	95.6
47/44	79.7	93.4	94.7	79.7	79.8	95.1	93.7	99.0	99.7	93.5	93.6	99.3
45	82.3	96.2	96.9	82.3	81.9	96.9	77.0	93.8	94.6	77.3	77.3	95.1
46	85.9	95.7	96.7	87.0	87.3	97.6	44.9	92.1	94.4	44.9	45.0	94.9
48	74.1	90.2	92.1	73.7	74.4	93.7	81.5	93.7	94.8	81.8	81.8	96.4
49	85.7	94.6	95.8	85.5	84.7	96.5	81.2	93.9	95.4	81.7	81.4	95.5
50	88.7	95.2	96.1	87.3	87.8	96.1	81.9	92.9	94.3	81.4	81.9	95.3
51	84.7	91.8	94.5	77.8	83.3	94.5	80.8	95.5	95.0	79.9	80.4	95.5
52	86.7	96.9	97.8	86.4	87.1	98.2	81.4	94.7	95.0	81.4	81.9	96.3

(Continued on following page)

Table S2. Alignment of protein-coding regions of EHVs in *Alphaherpesvirinae* (%)

ORF	Protein						DNA					
	1/4	1/8	1/9	4/8	4/9	8/9	1/4	1/8	1/9	4/8	4/9	8/9
53	91.1	97.5	98.2	89.9	90.3	98.1	83.5	94.6	95.0	83.7	83.8	96.4
54	79.1	92.5	95.1	78.6	79.4	96.1	78.7	91.8	93.6	78.6	78.7	95.5
55	84.4	94.7	96.4	83.4	84.8	96.7	80.6	92.8	93.3	79.8	80.4	96.4
56	89.2	95.8	96.7	89.4	88.5	97.0	83.4	92.9	94.3	83.3	83.4	95.7
57	92.6	96.3	97.0	91.5	92.0	97.0	84.2	94.3	93.3	84.1	83.5	94.8
58	83.9	92.3	92.4	83.3	82.1	95.0	79.8	90.6	92.4	79.9	80.1	94.2
59	69.9	84.4	87.2	66.5	67.0	90.7	74.0	87.4	88.7	72.6	73.3	92.3
60	89.1	96.7	99.1	87.7	88.6	97.6	84.7	94.1	95.0	84.6	85.5	95.9
61	78.5	91.0	93.6	77.0	77.3	93.9	75.9	91.5	92.9	76.2	75.6	93.5
62	74.2	89.2	97.3	80.7	80.0	90.8	79.2	87.3	91.6	81.2	79.2	89.4
63	58.5	85.3	88.0	57.8	59.3	86.1	69.0	89.2	91.1	70.1	69.8	92.1
64	83.3	86.8	95.3	76.8	84.6	89.3	82.2	92.6	95.1	81.3	82.8	94.4
65	85.2	96.2	97.3	86.3	85.9	97.2	83.4	94.3	95.4	83.4	82.8	94.8
66	80.3	94.0	95.7	81.6	81.1	94.4	79.3	92.6	95.5	79.1	79.5	93.9
67	67.7	93.8	94.8	68.0	67.9	96.7	65.8	93.9	95.7	66.6	66.5	95.8
68	59.2	80.9	79.7	68.8	65.2	93.1	63.1	91.5	91.1	67.7	66.4	93.7
69	86.9	92.9	95.3	85.6	85.6	93.7	81.7	92.3	94.3	81.6	80.6	93.1
70	72.4	92.2	92.0	72.9	71.0	92.5	75.9	91.5	92.6	76.3	75.9	94.1
71	59.0	92.1	88.7	50.4	59.8	86.0	68.6	96.9	92.3	63.7	68.5	90.9
72	76.6	91.0	96.5	77.6	74.6	91.3	78.2	91.1	96.7	80.0	78.3	92.3
73	73.8	90.6	94.8	72.1	73.8	92.0	75.6	92.9	93.6	75.1	75.9	92.7
74	85.4	96.0	97.8	86.3	85.6	97.5	81.3	94.9	96.9	81.1	81.6	96.0
75	61.1	85.4	96.9	63.0	61.1	84.6	60.1	86.8	96.9	61.0	61.0	87.0
76	53.5	82.2	88.6	52.6	52.6	86.3	59.5	87.1	91.7	58.7	59.4	90.8
